# A Retrospective Analysis of Characteristic Features of Responders and Impaired Patients to a Single Injection of Pure Platelet-Rich Plasma in Knee Osteoarthritis

**DOI:** 10.3390/jcm10081748

**Published:** 2021-04-17

**Authors:** Cécilia Bec, Axelle Rousset, Thibault Brandin, Pauline François, Sitraka Rabarimeriarijaona, Chloé Dumoulin, Gaëlle Heleu, Fanny Grimaud, Julie Veran, Guy Magalon, Françoise Dignat-George, Florence Sabatier, Marie-Laure Louis, Jérémy Magalon

**Affiliations:** 1 Cell Therapy Laboratory, Hôpital de la Conception, AP-HM, INSERM CIC BT 1409, 13005 Marseille, France; cecilia.bec@ap-hm.fr (C.B.); axelle.rousset@ap-hm.fr (A.R.); Thibault.BRANDIN@ap-hm.fr (T.B.); pauline.francois@ap-hm.fr (P.F.); Sitraka.RABARIMERIARIJAONA@ap-hm.fr (S.R.); chloe.dumoulin@ap-hm.fr (C.D.); Gaelle.HELEU@ap-hm.fr (G.H.); fanny.grimaud@ap-hm.fr (F.G.); julie.veran@ap-hm.fr (J.V.); florence.sabatier@ap-hm.fr (F.S.); 2 Aix Marseille Univ, INSERM, INRA, C2VN, 13005 Marseille, France; Francoise.dignat-george@univ-amu.fr; 3 Remedex SAS, 13008 Marseille, France; g.magalon@gmail.com; 4 Hematology and Vascular Biology Department, Hôpital de la Conception, AP-HM, 13005 Marseille, France; 5 Orthopedic Department, Hopital Nord, AP-HM, 13015 Marseille, France; MarieLaure.LOUIS@ap-hm.fr; 6 ICOS, Sport and Orthopedics Surgery Institute, 13008 Marseille, France; 7 Orthopedic Department, Clinique Juge, Almaviva, 13008 Marseille, France

**Keywords:** platelet-rich plasma, knee osteoarthritis, biological characterization, KOOS score, growth factors

## Abstract

(1) Background: The emergence of injectable “biologic” medication creates a new approach to treat osteoarthritis (OA). Among them, the use of intra-articular injection of PRP became widespread despite the absence of consensus regarding its optimal composition. The aim of this study was to retrospectively correlate an extensive biological characterization of injected PRP to the clinical responses of patients presenting knee OA. (2) Methods: This retrospective study included 75 patients with knee OA. Cartilage lesions were assessed using magnetic resonance imaging and the International Cartilage Regeneration Society (ICRS) classification. PRP extensive biological characterization was performed and patients’ subjective symptoms were recorded before injection and 3 and 6 months after injection using the Knee injury and Osteoarthritis Outcome Score (KOOS). Responders were defined by an improvement of 10 points on KOOS. (3) Results: At 6 months, 63.0% of the patients were responders. Impairment was characterized by a significantly higher proportion of patients with three compartments altered at baseline MRI and receiving a significantly higher dose of platelets compared to responders. (4) Conclusions: Single injection of pure PRP resulted in significant clinical improvement in the management of knee OA. Both baseline MRI and PRP biological features may be predictive factors of the clinical response, highlighting that a better understanding of action mechanism of PRP is still required.

## 1. Introduction

Osteoarthritis (OA) is a degenerative joint pathology of the cartilage. This highly prevalent multifactorial musculoskeletal disorder represents a substantial burden for the health-care system [1].

According to the Global Burden of Disease study published in 2017, three hundred million people are suffering from OA worldwide and its incidence is constantly increasing due to the aging of the population and the traumas associated with the development of sports practices [2]. OA is a multifactorial disease where genetic, hormonal, age, mechanical and metabolic factors interact and lead to deterioration and loss of articular cartilage associated to structural and functional changes in the joint [3]. Knee osteoarthritis (KOA) represents approximately 89% of the burden of OA worldwide [2]. The diagnosis of KOA is mainly performed by clinical examination seeking pain and swelling of the knee, considered as the main symptoms. Confirmation is generally accomplished with radiography [4]. First-line treatment for KOA is purely symptomatic and the essential goal is to relieve pain. Usually, a combination of non-pharmacological approaches such as dietary supplements, muscle strengthening exercises and pharmacological therapies (including analgesics, non-steroid anti-inflammatory (NSAI) drugs and corticosteroid or hyaluronic acid (HA) injections) is prescribed [5]. However, this provides only temporary benefits and could be associated with side effects for corticosteroids especially a decrease cartilage volume, systemic effects (hyperglycemia, warmth, flushing of the skin) and risk of subsequent infections [6,7].

Recently, the emergence of injectable “biologic” medication constitutes a new approach to treat OA [8]. Although not formally defined, this umbrella term includes innovative products obtained either after a single step procedure (platelet-rich plasma (PRP), adipose-derived stromal vascular fraction, bone marrow aspirate concentrate) or following several weeks of cell culture (autologous adipose or bone marrow derived mesenchymal stem cells (MSC)). Among these products, the PRP is experiencing increasing popularity in various fields, especially in KOA [9]. This biological drug is defined as an autologous plasma suspension of platelets characterized by a higher platelet concentration than peripheral blood [10]. Once injected, platelets are activated by physiological activators (collagen, calcium) and release high levels of growth factors (GFs) involved in reparative and regenerative processes including platelet-derived growth factors (PDGFs), transforming growth factor β1 (TGF-β1), vascular endothelial growth factor (VEGF), epidermal growth factor (EGF), insulin-like growth factor 1 (IGF-1), or fibroblast growth factor (FGF) [11,12]. These GFs act at various levels to restore the joint homeostasis as reported by several pre-clinical models describing chondrocytes anabolism and chondral remodeling, increased HA secretion and down-regulation of inflammation and apoptotic pathways following PRP injections [13,14].

Thus, PRP, delivered intra-articularly, appears to be an innovative autologous cell therapy product for the treatment of cartilage lesions and several reviews have proved its efficiency in this indication. However, one of the main weaknesses in this field limiting the credibility of PRP use by a larger cohort of physicians is the lack of a precise biological characterization of the content of the PRP injected, thus hampering the understanding of the mechanism associated with PRP efficacy. Chahla et al. [15] reviewed about 100 studies and reported that more of 80% of them did not provide accurate details about the production protocol and the resulting PRP, making comparison between studies unreliable. Indeed, substantial differences in the content of platelets concentrates produced by the various automated and manual protocols have been described [16,17]. The impact of such differences in PRP characteristics on the regenerative processes of different tissues have been suggested and their consequences on clinical results in PRP therapy is a hot topic [18,19].

The aim of this study was to analyze the response of patients presenting KOA and treated with a single PRP in routine care in a university hospital and to investigate the relationships between clinical results and PRP composition by conducting a precise biological characterization of the administered PRP, including quantification of growth factors.

## 2. Materials and Methods

### 2.1. Participant Recruitment

This retrospective observational study was performed at a single university orthopedic department (La Conception Hospital, Marseille, France). Patients with established KOA based on radiographic Kellgren and Lawrence (KL) scale were addressed to our university center for PRP injection. Patients included in the study had magnetic resonance imaging (MRI) examination at baseline and a Knee injury and Osteoarthritis Outcome Score (KOOS) questionnaire available at initiation andat least at 3-month or 6-month follow-up. Exclusion criteria were intra articular knee injection of corticosteroid less than 8 weeks before inclusion; intra articular knee injection of hyaluronic acid less than 8 weeks before inclusion; Aspirin, platelet inhibiting agent, antivitamin K or NSAI treatment completed less than 2 weeks before inclusion, infectious disease or positive serology to VIH-1, HCV, HBV and syphilis. All patients provided informed consent and all procedures were performed in accordance with the 1964 Helsinki Declaration and its later amendments.

MRI examination was performed on Magnetom Aera 1.5 T Siemens MRI scanner (Siemens Healthcare GmbH, Erlangen, Germany) with a 15-channels transmitter/receiver knee coil with patients in supine position knee extended. Assessments were performed by a senior orthopedist in charge of the follow-up of the patients. The femoro-tibial (medial and lateral) and femoro-patellar compartments were assessed. Affected compartments corresponding to grades II, III and IV were reported before the injection according to the International Cartilage Regeneration Society (ICRS) MRI classification [20].

### 2.2. Evaluation Tools and Follow-Up

The patient’s subjective symptoms were prospectively assessed at baseline, 3 and 6 months after injection, using KOOS score. This is a benchmark score in the clinical evaluation of various knee pathologies. It contains 42 items divided into 5 categories: symptoms, pain, activities of daily living (ADL), sports and recreation function, quality of life (QOL). A percentage ranging from 0 to 100% is obtained, 0% representing extreme knee problems and 100% representing no knee problems. Hip Knee Ankle (HKA) alignment was assessed for all patients at baseline.

Responders to PRP injection were defined as patients presenting an improvement compared to baseline of at least 10 points in KOOS total score which correspond to the Minimum Clinically Important Difference (MCID). Impaired patients were defined as patients presenting an impairment of at least 1 point compared to baseline in KOOS total score.

### 2.3. Platelet-Rich Plasma Preparation

After a four-step skin decontamination (antiseptic foaming solution, rinsing with sterile water, drying and alcoholic dermal antiseptic), 18 mL of peripheral blood was collected by venepuncture using a 21-gauge needle filling one 20 mL syringe containing 2 mL of anticoagulant citrate dextrose solution-A (ACD-A) (Fidia, Abano Terme, Italy). The blood was transferred into the Hy-tissue 20 PRP device (Fidia, Abano Terme, Italy) before centrifugation using the Omnigrafter 3.0 (Fidia, Abano Terme, Italy) and PRP Large Volume Cycle (3200 rpm during for 10 min). All plasma was recovered using a 20 mL syringe through the Push-out system (Fidia, Abano Terme, Italy).

### 2.4. Biological Parameter Quantification

#### 2.4.1. Blood and PRP Cell Counting

A volume of 300 µL from whole blood and each PRP preparation were sampled to determine platelets, leukocytes and red blood cells concentrations and platelets relative parameters (Immature Platelet Fraction (IPF), mean platelet volume (MPV)) using automated hematology blood cell analyzers Sysmex XN-10 (Sysmex, Kobe, Japan), in accordance with recently published guidelines [21]. The IPF is the proportion of immature platelets in the blood. Immature platelets are the youngest platelets resulting from the fragmentation of megakaryocytes and released directly into the bloodstream.

#### 2.4.2. Growth Factors Release Measurement

Activation of PRP with CaCl_2_ was performed to induce GF release and supernatants were frozen at −20 °C before quantification. A combination of 12 cytokines and growth factors (GFs) classified as inflammatory (Interleukin-1β, Tumor Necrosis Factor-α (TNF-α), Interleukin-6 (IL-6), Inteferon γ (IFN γ)), anti-inflammatory (Interleukin-1 Receptor antagonist (IL1-Ra), Interleukin-10 (IL-10)) and regenerative (Platelet Derived Growth Factor (PDGF) AA-BB or AB-BB, Vascular Endothelial Growth Factor (VEGF), Epidermal Growth Factor (EGF), Fibroblast Growth Factor 2 (FGF2), Transforming Growth Factor β1 (TGF-β1)) were measured using a Magpix instrument (Luminex xMAP Technology, Luminex Inc., Austin, TX, USA) allowing simultaneous measurement of the different analytes in small sample volume.

#### 2.4.3. Microbiological Assay

The 250 µL of PRP were sampled in Bactec culture bottles (Peds Plus Aerobic/F and Plus Anaerobic/F culture vials, containing each 40 mL of medium). The Bactec method (Becton Dickinson, Sparks, MD, USA) uses a computer-controlled incubation/detection system. The media used contained proprietary factors designed to inactivate a wide variety of antibacterial and antifungal agents. Bactec culture bottles were incubated at 37 °C for a total of 10 days and automated readings were taken every 10 min. Detection of organisms resulted in an audible alarm and automatic recording of time to detection.

### 2.5. Injection

The intra-articular knee injection was performed, after a four-step skin decontamination (antiseptic foaming solution, rinsing with sterile water, drying and alcoholic dermal antiseptic), with a 21-Gauge needle by a senior orthopedic surgeon without ultrasound assistance. Pain visual analog scale (VAS) upon injection was assessed.

### 2.6. Statistical Analysis

All data are presented as the mean ± standard deviation. Data were analyzed with GraphPad Prism (GraphPad Software, La Jolla, CA, USA).

Baseline characteristics of both groups were compared using a Ӽ-square test or Student *t* test according to the nature of the variables. Mean differences between biological characteristics of all PRP were compared using a nonparametric Mann–Whitney *t* test two tailed. While we used a nonparametric Mann–Whitney *t* test one-tailed to compare patient responder and other. We also used a two-way analysis of variance to compare the KOOS Score at different time between responder and impaired state after treatment. Statistical significance was accepted for *p* values < 0.05.

Biological PRP characteristics were described and calculated using hematologic cell counts and GF quantification. The increase factors in platelets or leukocytes were obtained by dividing the concentration of the cells in question in PRP by the concentration in whole blood. Doses of platelets or GFs were obtained by multiplying the volume of PRP injected by the corresponding concentration. We also assessed immature platelets factor, mean platelet volume and dose of immature platelets to characterized platelets in the product.

The correlation between the mean platelet volume and the dose of immature platelets with all the dose of the growth factor was analyzed.

## 3. Results

### 3.1. Patient Characteristics

A total of 75 patients with KOA, aged from 32 to 82 years (61 ± 16 years) were enrolled between November 2017 and October 2020, corresponding to a total of 75 injections. Among them, 48 were female (64%) and 27 were male (36%). Mean body mass index (BMI) was 26.74 ± 3.72 kg/m^2^. Previous injection of hyaluronic acid or PRP were reported in 82.7% and 26.7% of the patients, respectively. Mean KOOS score at baseline was 40.9 ± 16.2 with a major impact on sport (23.9 ± 21.6) and quality of life (22.9 ± 19.3) subscales. Pain, symptoms and activity in daily living were less severe with a mean score at 50.2 ± 18.2, 53.0 ± 20.7 and 53.9 ± 20.2, respectively. Radiographic evaluation revealed that majority of the patients had a grade II (22 patients; 29.0%) or III (38 patients; 51.5%) KOA according to KL scale whereas 5 (6.5%) and 10 (13.0%) patients presented a grade I or IV, respectively. Analysis of cartilage lesions using MRI revealed that 27.0%, 43.3% and 29.7% of patients presented either one, two or three compartments with at least grade II OA according to the ICRS classification. Furthermore, 48.5% of the patient had at least one compartment reaching a grade IV. Hip knee ankle alignment was normal for 45.3% of the patients whereas 20.0% and 26.7% had varus or valgus deformity.

### 3.2. Biological Characteristics of PRP Injected

The results of the extensive biological characterization performed on each PRP injected are listed in Table 1. The injected volume of PRP ranged between 5 and 9 mL. The mean platelets purity is higher than 95% (95.46 ± 2.01) with a percentage of red blood cells (RBC) and leucocytes of 4.35 ± 1.96 and 0.19 ± 0.14, respectively. The total dose of platelets injected was 2.87 ± 0.88 billion. The preparation process leads to the obtention of PRP with good reproducibility regarding platelets purity, increase factor in platelets and recovery rate with coefficient of variation (CV) below 20% whereas other biological parameters are more variable. Measurements of soluble factors secreted after PRP activation revealed the presence of highly variable concentration of regenerative growth factors (CV between 29.8 and 101.3%), whereas only one from the three anti-inflammatory and inflammatory cytokines were detectable at a significant level (TNF-α and IL1-Ra).

### 3.3. Evolution of the KOOS Score

A statistically significant improvement of 16.68 +/− 16.08 (*p* < 0.0001) and 17.33 +/− 20.71 (*p* < 0.0001) points in KOOS score was observed at 3 and 6 months, respectively. This significant difference was also observed in pain, symptoms, activity in daily living and quality of life at both follow-up appointments. Significant improvement in sports subscale was also observed at 3 months (*p* = 0.002). The proportion of responder patients was stable at 63%, between 3 (44 patients) and 6 (34 patients). Conversely, the proportion of impaired patients slightly increased from 15.7% (11 patients) to 20.4% (11 patients). Other patients were improved without reaching the MCID of the KOOS score.

### 3.4. Comparison of Responders and Impaired Patients Characteristics

The two groups were similar in terms of age, sex ratio and numbers of and injected volume of PRP (Table 2). KOOS score at baseline was statistically higher in responders compared to impaired patients (*p* = 0.02). This significant difference is also retrieved in “sport and recreation function” and “quality of life” subscales. Baseline radiographic and MRI analysis revealed statistical differences between the responders and impaired patients. No patients with severe KOA (grade IV) according to the KL scale were responders whereas they represented 45.4% of the impaired patients (*p* < 0.0001). MRI analysis showed that the number of joint compartments altered (*p* = 0.01) was significantly different with a proportion of patients with three compartments altered higher in the impaired group.

**Figure 1:**
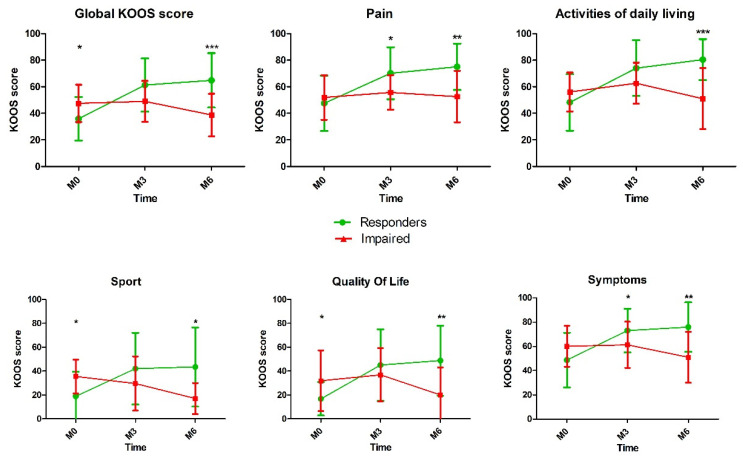
Comparison of Knee injury and Osteoarthritis Outcome Score (KOOS) score after platelet-rich plasma (PRP) injection between responders and impaired patients (*, *p* < 0.05; **, *p* < 0.01; *** *p* < 0.001).

As expected, KOOS total score and all subscales showed a statistical difference between the two groups at 3 and 6 months. The evolution of the KOOS score and subscales over the time is reported in the Figure 1.

**Figure 2:**
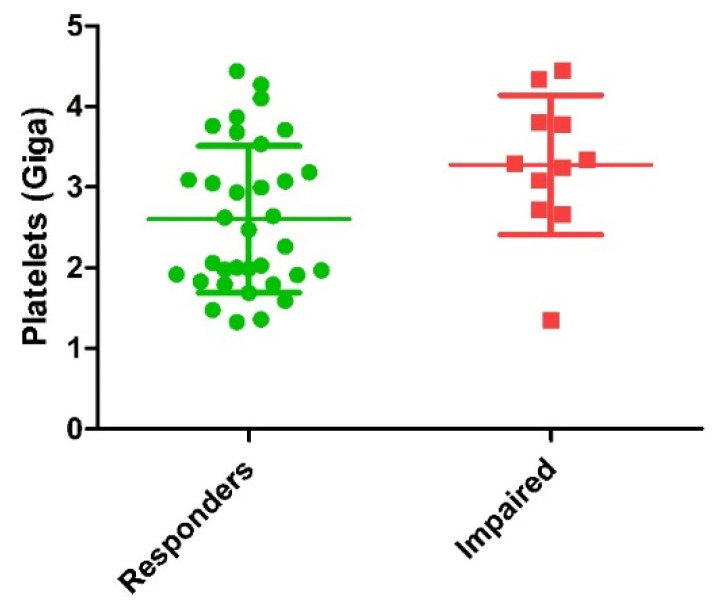
Comparison of dose of platelets injected between responders and impaired patients (*p* = 0.01). Green circles represent responder’s patients and red squares represent impaired patients.

Comparison of biological characteristics of PRP injected between responders and impaired patients is detailed in Table 3. Main finding was the significantly higher dose of platelets injected in the impaired patients’ group (Figure 2). Mean dose of growth factors and cytokines potentially delivered were all higher in the impaired groups with a significant difference for VEGF (*p* = 0.02) and a trend for TGF-β1 (*p* = 0.16), EGF (*p* = 0.09) and IL1-Ra (*p* = 0.10). Other biological parameters were not statistically different between the two groups.

## 4. Discussion

A single administration of autologous pure PRP provided significant clinical benefit to more than 60% at the three and six months, in patients presenting KOA that affects one to three knee compartments with grade II to IV OA according ICRS MRI classification.

Interestingly, the responders’ rate is lower compared to the recent study conducted by Guillibert et al. reaching 80% of responders six months after the injection using the same preparation method [22]. However, this study differs from the current one on several points: (i) the responder’s definition was not only based on KOOS score but using the OMERACT OARSI criteria (Outcome Measures in Rheumatology, Osteoarthritis Research Society International); (ii) severity of KOA was targeted on grade II/III defined using radiological KL scale; (iii) the full PRP volume was systematically recovered leading to mean final volume injected of 8.8 mL. This aspect is of importance as knee injection should take into account of the articular capacity of the knee in order to favor a better distribution of PRP throughout the joint. It was recently recommended that the volume for knee-specific injection should be at 9 mL [23] reinforced by a comparative study comparing different PRP volumes injection concluding that optimum therapeutic volume of PRP was 10 mL [24]. Regarding PRP quality, both studies used a pure PRP formulation based on in vitro data describing exposition of synovial cells with leucocyte-rich PRP and RBCs resulting in significant cell death and proinflammatory mediator production [18]. The scientific rational to avoid RBCs in final PRP is based on the potential release of hemoglobin, hemin and iron caused by RBCs lysis, which can induce oxidative stress and pro-inflammatory reactions in tissues [25]. Additionally, a recent meta-analysis reported that leukocyte-poor PRP may be a superior line of treatment for KOA over leukocyte-rich PRP leading to a consensus of leukocyte-poor, RBC-free formulations of PRP when intra-articularly administered. From a broader clinical perspective, these results are consistent with the recent meta-analysis results from Belk et al. indicating that patients undergoing PRP injection for KOA with PRP can be expected to experience improved clinical outcomes when compared with hyaluronic acid [19].

This study provides radiological, clinical and biological data whose goal is to identify predictive factors of efficacy. In order to facilitate the comprehension, we decided to focus our comparison between responders and impaired patients based on change in total KOOS score 6 months after the injection.

Interestingly, radiographic and MRI characteristics showed a significant difference between the two groups as impaired patients presented higher proportion of patients with severe KOA according to the KL scale with three knee joints compartments altered. Although not significant, the proportion of patients with at least one compartment with grade IV OA according ICRS classification was 73% in impaired patients compared to 46.7% in responders’ groups which could also influence the clinical results. To our knowledge, whether which MRI parameter according ICRS classification from the number of affected compartments or the severity of the grade is better correlated with clinical settings remains a question which has never been studied. Surprisingly, MRI data were not correlated with the baseline clinical setting as KOOS score reported by impaired patients was significantly higher. This is in line with findings reporting that KOOS scores are poor indicators and weakly correlated with tibiofemoral cartilage loss [26]. These findings also suggest that establishing a specific PRP injection protocol (multiple injections, mix with hyaluronic acid) for severe KOA is an area that deserves to be studied.

The major interest of this retrospective study is the application of the American Academy of Orthopedic Surgeons (AAOS) recommendations by performing a rigorous biological characterization of the whole blood and PRP for each injection and by setting up a post treatment monitoring [27]. This is also in line with and the Minimum Information to provide for studies evaluating Biologics in the Orthopaedics field also called MIBO [28]. The goal of these recommendations is to facilitate clinical and experimental investigations in order to elucidate and precise the mechanisms of action supporting PRP efficacy in a broad range of diseases. That is why we also performed an exhaustive biological characterization of PRP injected including original biological parameters never reported inside PRP preparation. Among them, the measurement of the immature platelet fraction (IPF) [29], which represents a population of newly formed platelets containing a greater amount of residual RNA and mean platelets volume (MPV) appeared to be a good candidate as efficacy predictive factor as they should be correlated with GF quantity. Unfortunately, none of them was correlated with clinical outcomes.

One interesting finding was the platelets dose injected which was found to be higher in the impaired patients group compared to the responders one. The impact of platelets concentration on PRP efficacy is a recurrent topic in this field. A recent review analyzed the in vitro effect of PRP on proliferation of human cells in vitro and reported that when the PRP ratio to media ratio increased, the proliferation rate decreased [30]. The in vitro study conducted by Graziani et al. reported the effect of varying PRP concentration on the proliferation of fibroblasts and osteoblasts. The authors conclude that the maximal PRP concentrations used does not provide the optimal environment for the promotion of wound healing [31]. The higher dose of platelets injected in impaired patients might result in excessive GF release as impaired patients presented a mean dose of GF potentially delivered higher than the responder patients, with a significant difference concerning VEGF. The potential negative effect of VEGF high doses in KOA seems surprising, although high VEGF expression in bone marrow lesions was associated with persistent pain in patients with hip OA [32]. However, from a clinical perspective, a recent controlled study using a standardized volume of leukocyte-poor PRP (8 mL) and high dose of platelets (10 billion) provided a long sustained chondroprotective effect up to one year in moderate knee OA higher than HA [33].

These contradictory results underline that the exact mechanism of action of the PRP is still unknown and the role of each growth factor is poorly understood. AAOS reported that many of the growth factors and cytokines present in PRP act within opposing biologic pathways and may have a beneficial effect in certain applications and deleterious effects in others. Interestingly, we previously reported in randomized controlled trial that doses of TGF-β1 was correlated to the quantity of injected platelets and higher in non-responder patients [34]. In our study, this difference is not significant, but a trend (*p* = 0.16) shows higher TGF-β1 dose in impaired patients. Indeed, scientific literature provides pre-clinical data supporting side effects of TGF- β 1 on mice and rabbit models including stimulation of synovial stimulation and fibrosis, attraction of inflammatory leukocytes to the synovial lining and induction of osteophyte formation [35,36]. In other musculoskeletal conditions, profibrotic effect of TGF- β 1 might be beneficial in tendon and ligament healing but may cause a deleterious effect in the healing of muscle injury [37,38]. Thus, understanding the role of each growth factor in the development of specific disease processes will facilitate the identification of therapeutically relevant components of PRP for each indication and the development of customized PRP preparations most suited to the specific indication.

Our study had limitations, including the absence of a control group which is an important weakness knowing the substantial placebo effect in arthrosis care [39] and a retrospective design associated with missing data for some scores. Finally, MRI follow-up six months after the injection could have been informative to complete the evaluation of the PRP efficacy.

In conclusion, our study provided further insights towards a better understanding of the biological activity of PRP in KOA. We showed that a single injection of pure PRP could provide a significant clinical improvement in the management of KOA 6 months after the injection. Both baseline radiographic/MRI and PRP biological features may be predictive factors of the clinical response reinforcing the need to follow recent AAOS guidelines about the importance to report detailed procedure when performing PRP injection and to set-up registries for post-marketed monitoring.

## Figures and Tables

**Table 1:** Mean biological characteristics of injected platelet-rich plasma.

Biological Characteristics	Mean ± SD	Min-Max	Coefficient of Variation (%)
Injected Volume (mL)	6.77 ± 1.43	5.00–9.00	21.04
Increase factor in platelets	1.82 ± 0.30	0.86–2.65	16.35
Increase factor in leucocytes	0.16 ± 0.12	0.003–0.540	76.20
Recovery rate in platelets (%)	85.15 ± 10.05	62.30–103.00	11.80
Sterility (%)	100	100	0
Relative composition			
Platelets (%)	95.46 ± 2.01	89.40–98.67	2.11
Leucocytes (%)	0.19 ± 0.14	0.01–0.58	73.27
Red blood cells (%)	4.35 ± 1.96	1.31–10.07	45.03
Absolute composition			
Platelets dose injected (billion, 10^9^)	2.87 ± 0.88	0.91–4.45	30.63
Leucocytes dose injected (million, 10^6^)	6.06 ± 5.44	0.10–27.65	52.57
Red blood cells dose injected (billion, 10^9^)	0.13 ± 0.07	0.05–0.33	53.48
Platelets relative parameters			
Immature Platelet Fraction (%)	3.44 ± 1.83	1.00–9.00	53.20
Immature platelets (billion, 10^9^)	0.10 ± 0.06	0.02–0.27	52.57
Mean platelets volume (fL)	10.14 ± 0.68	8.80–12.00	53.48
Growth factors *			
Inflammatory			
TNF-α (pg)	141.6 ± 68.56	35.75–387.8	48.41
IL-6 (pg)	Not detectable	Not detectable	Not detectable
IFN-g (pg)	Not detectable	Not detectable	Not detectable
Anti inflammatory			
IL-1Ra (pg)	164.0 ± 267.9	0.0–1125.0	163.34
IL-10 (pg)	Not detectable	Not detectable	Not detectable
Regenerative			
PDGF AB BB (fg)	228.4 ± 100.4	69.6 ± 469.3	43.94
VEGF (pg)	451.6 ± 421.6	0.0–1476	93.34
EGF (pg)	1231 ± 721.0	138.8–4334	58.59
FGF-2 (pg) **	1157 ± 1173	0.0–4164.0	101.34
TGF-β1 (fg)	361.2 ± 107.6	154.3 ± 675.1	29.79

* performed on 43 patients; ** performed on 22 patients; TNF-α: tumor necrosis factor-α, IL: interleukin, IFN-g: Interferon gamma, IL1Ra: interleukin-1 receptor antagonist, PDGF: platelet-derived growth factor, VEGF: vascular endothelial growth factor, EGF: epidermal growth factor, FGF2: fibroblast growth factor 2, TGF-β1: transforming growth factor β1.

**Table 2:** Baseline characteristics of responders and impaired patients.

	Responders (*n* = 34)	Impaired (*n* = 11)	*p*-Value
Age	61.00 ± 11.00	66.73 ± 7.00	0.09
Sex Ratio (H/F)	12/22	2/9	0.46
Body Mass Index (kg/m^2^)	26.36 ± 3.60	27.24 ± 2.98	0.44
Previous HA injection: *n* (%)	28 (82.0)	8 (82.0)	1
Previous PRP injection: *n* (%)	8 (23.5)	4 (36.4)	0.06
Volume injected	6.50 ± 1.53	6.36 ±1.57	0.40
Baseline KOOS score	35.97 ± 16.42	47.55 ± 14.07	0.02
Pain	47.59 ± 20.89	52.36 ± 16.77	0.54
Symptoms	48.74 ± 22.51	60.09 ± 16.80	0.15
ADL	48.29 ± 21.29	16.09 ± 14.62	0.27
Sport	18.85 ± 20.62	35.50 ±14.23	0.01
QOL	16.82 ± 14.05	31.90 ± 25.44	0.03
Kellgren and Lawrence scale (%)			
I	17.7%	0%	
II	17.7%	18.2%	<0.0001
III	64.7%	36.4%	
IV	0%	45.4%	
MRI characteristics according ICRS classification			
Proportion of patient presenting:			
One compartment affected	11.5%	3.8%	0.01
Two compartments affected	49.1%	38.5%	
Three compartments affected	39.3%	57.7%	
Proportion of patients with at least one compart-ment presenting grade IV OA:	46.7%	73.0%	0.17
Pain at the injection (VAS)	27.88 ± 27.36	32.73 ± 24.12	0.55

HA: hyaluronic acid, PRP: Platelet-rich plasma, ADL: activities of daily living, QOL: quality of life.

**Table 3:** Comparison of mean biological characteristics of PRP from responders and impaired patients.

Biological Characteristics	Responders (*n* = 34)	Impaired (*n* = 11)	*p*
Injected Volume (mL)	6.51 ± 1.53	6.36 ± 1.57	0.40
Increase factor of platelets	1.76 ± 0.33	1.87 ± 0.11	0.08
Increase factor of leukocytes	0.13 ± 0.11	0.19 ± 0.15	0.11
Relative composition			
Platelets (%)	95.42 ± 1.76	95.87 ± 1.89	0.31
Leukocytes (%)	0.16 ± 0.12	0.20 ± 0.19	0.49
Red blood cells (%)	4.41 ± 1.74	3.93 ± 1.74	0.32
Absolute composition			
Platelets (10^9^)	2.60 ± 0.91	3.28 ± 0.87	0.01
Leukocytes (10^6^)	4.80 ± 5.27	7.03 ± 6.12	0.10
Red blood cells (10^9^)	0.108 ± 0.052	0.123 ± 0.087	0.42
Platelets relative parameters			
Immature Platelet Fraction (%)	3.33 ± 1.79	2.87 ± 1.72	0.19
Immature platelets (10^9^)	0.08 ± 0.05	0.09 ± 0.07	0.28
Mean platelet volume (fL)	10.21 ± 0.70	9.80 ± 0.72	0.08
Growth factors	*n = 25*	*n = 8*	
Inflammatory			
TNF-α (pg)	123.4 ± 51.3	162.0 ± 96.3	0.22
IL-6	Not detectable	Not detectable	Not detectable
IFN-g	Not detectable	Not detectable	Not detectable
Anti inflammatory			
IL-1Ra (pg)	113.9 ± 179.9	378.3 ± 451.5	0.05
IL-10 (pg)	Not detectable	Not detectable	Not detectable
Regenerative			
PDGF AB BB (fg)	216.6 ± 81.3	241.3 ± 131.9	0.29
VEGF (pg)	401.8 ± 345.1	803.5 ± 445.0	0.008
EGF (pg)	1068.0 ± 607.4	1453.0 ± 368.9	0.05
FGF-2 (pg)	1026.0 ± 746.5	1200 ± 1726	0.32
TGF-β1 (fg)	354.8 ± 103.5	397.1 ± 118.7	0.16

TNF-α: tumor necrosis factor-α, IL: interleukin, IFN-g: Interferon gamma, IL1Ra: interleukin-1 receptor antagonist, PDGF: platelet-derived growth factor, VEGF: vascular endothelial growth factor, EGF: epidermal growth factor, FGF2: fibroblast growth factor 2, TGF-β1: transforming growth factor β1.

## Data Availability

Raw data under excel format can be provided on demand.
